# Coproducing an Ecological Momentary Assessment Measurement Burst Mental Health Study With Young People: The MHIM Coproduction Protocol

**DOI:** 10.1111/hex.70218

**Published:** 2025-03-11

**Authors:** Luke Power, Tong Xie, Thomas Bartlett, Dejla Hoxha, Heather Bryson, Eva Drummond, Poppy Fairbairn, Alex Swan, Yu‐Wen Tan, Lorna Caddick, Aja Murray

**Affiliations:** ^1^ School of Philosophy, Psychology and Language Sciences, Psychology Department University of Edinburgh Edinburgh Scotland; ^2^ Faculty of Psychology Beijing Normal University Beijing China; ^3^ School of Social and Political Sciences, Social Work Department University of Edinburgh Edinburgh Scotland; ^4^ Expert by Experience, Member of pre‐study YPAG Scotland; ^5^ Division of Psychiatry, Centre for Clinical Brain Sciences University of Edinburgh Edinburgh Scotland

**Keywords:** adolescent mental health, coproduction, longitudinal study, protocol

## Abstract

**Background:**

There is now widespread recognition within adolescence mental health research of the ethical imperative and benefits of coproduction. This has led to the development of best practice guidelines and the routine reporting of coproduction methods. However, there are unique considerations associated with involving young people in research with varying designs, e.g., longitudinal. Ideally, these decisions are made in consultation with young people themselves. Thus, the objective and novel contribution of this paper is (1) the involvement of young people in the development of the MHIM protocol, including an evaluative framework, and (2) a study protcol that utilises coproduction in the context of a longitudinal mental health project.

**Methods:**

We present a coproduction protocol (MHIM‐YPAG), developed with young people (pre‐study YPAG), that describes the planned approach to involving young people in the MHIM study. MHIM will use an accelerated longitudinal‐cohort design that will combine ecological momentary assessment, bio‐sampling, radar‐based sleep measurement and online questionnaires to examine the relationship between daily‐life‐experiences and adolescence mental health. The protocol describes how young people will be involved in MHIM and how impact will be evaluated. Within this context, coproduction relates to (1) the codevelopment, with young people, of the study's structure and (2) their involvement as researchers, e.g., recruitment, data interpretation and knowledge‐exchange.

**Patient or Public Contribution:**

As one of the first protocols of its kind, our coproduction protocol, informed by literature and consultations with young people (pre‐study YPAG), can provide a template for planning and reporting coproduction in mental health studies of adolescence.

## 1 Aim

Before an examination of relevant background literature, it is important to outline the primary aim of this paper. Its purpose is to present a protocol, including an evaluative framework for measuring MHIM‐YPAG success for those involved. This will be informed by both relevant literature and insights from young people.

## 2 Background

Within Western countries, the rate of mental ill‐health has been gradually increasing [1]. Adolescence is a particularly crucial period, with mental ill‐health typically manifesting before age 24 [2, 3, 4, 5]. The 2022 Health Behaviours in School‐Aged Children Report found that, in Scotland, 32% of young people experienced anxiety, 14% were at risk of depression, and 21% experienced low mood [6]. Given this, mental health research that focuses on the period defined as adolescence is critically important.

Alongside the need to examine the mental health of this population, there is increased recognition of the value and importance of including young people as coproducers. Aside from an ethical imperative to include young people in decisions affecting them, as per the United Nations Convention on the Rights of the Child (UN CRC), doing so has shown to benefit both young people and research projects alike [7]. Though demonstrating the impact of coproduction is difficult, evidence suggests it can enhance (1) recruitment and retention, (2) research tool relevancy, (3) outcome measures/interventions and (4) study acceptability/feasibility [8, 9, 10]. Consistent with this, a recent survey found that those who used coproduction in adolescent health research emphasised its benefits [11].

The engagement of young people in mental health research has tended to take the form of an advisory group that aims to generate research that is more acceptable, more relevant and better tailored to the target population [12, 13]. The example of the NeurOX Young Person Advisory Group (YPAG) highlights that YPAG involvement directly influences the research process, e.g., shifting research towards themes deemed more relevant and implementing more effective recruitment strategies [10]. YPAG members also indicated that it helped them develop technical and soft skills. While still under‐utilised [14], coproduction, specifically with young people, is beginning to be used in a diversity of mental health‐focused research projects [15, 16, 17, 18].

While beneficial, coproduction is typically underutilised due to the fact that it is time/resource intensive and requires the development of meaningful and trusting relationships, something which takes time [19]. In addition, due to funding constraints, most studies do not have the resources/time to do this. However, with the support of funders who understand the importance of coproduction, longitudinal designs are able to overcome these constraints.

Studies have also indicated limitations of involving young people as coproducers. For instance, Mandoh et al. [20] found that YPAGs are typically small and those who participate typically have past experience in coproduction. This, they argue, leads to a failure in representation. Poor representativeness can lead to the construction of biased programmes/tools/projects. The meaning of representation, how this is operationalised in research and finally how this impacts those who are involved in research is a critical consideration. Scholz et al. [21, 22] noted that those involved are typically framed as ‘community leaders’ and are thus depicted as representatives of an entire community. This, they argue, can lead to the downplaying of individual perspectives and subsequent disempowerment. Scholz et al. [21] argue that to avoid this, a reconceptualisation of representation is needed alongside the diversification of those involved. Thus, there is a need for research that includes a diverse range of young people.

## 3 Hierarchy of Involvement and the Diverse Methods of Involving Young People

Sellars et al. [23] present a 5‐level hierarchy to describe the different levels of involvement (see also [24, 25, 26]). The five levels are (1) affirmation, (2) light consultation, (3) interactive advice, (4) coproduction and (5) youth led. Sellars et al. [11] detail how these five‐levels are associated with four stages of the research process (see also [27]). These include research design, conducting, data analysis and output and dissemination. The involvement of young people can occur within all four stages and their level of involvement can vary. By allowing varying degrees of involvement, it is possible to manage some of the difficulties associated with young people's scheduling demands, e.g., those associated with school work.

While the choice of involving young people is project dependent, coproduction should be an integral aspect of research, specifically over consultation, as it has a number of practical benefits and is arguably the ethical choice. However, more research is needed on how to do it well. As discussed later, the methods by which our pre‐study YPAG members were engaged in developing this coproduction protocol is akin to interactive consultation. However, the MHIM‐YPAG that the protocol describes is a process of coproduction.

## 4 Current Best Practice Guidance on Involving Young People

Principles and guidelines have also been developed. INVOLVE outlined five core coproduction principles: sharing of power, building and maintaining relationships, including all perspectives and skills, respecting/valuing each other's knowledge and reciprocity [28]. In addition, The New Economics Foundation developed six principles of coproduction: taking an assets‐based approach, building on people's existing capabilities, reciprocity and mutuality, peer support networks, blurring distinctions, and facilitating over delivering [29]. Both sets of principles have been used as a guiding framework in shaping coproduction.

In terms of best practices, Wilson et al. [30] grouped five clusters: consent, evaluating risks and comparing against benefits, tailored communication, trust‐building and balanced power‐sharing, logistics of meetings. Hawke et al. [31] integrated their own experiences with young people's insights to provide a list of ‘do's and don'ts’ in relation to young people's involvement. Chan et al. [32] developed an online guidance tool to support setting up and operating YPAGs. Others have focused on reporting practices and have recommended frequency, methods and nature of involvement, parental involvement and recognition of involvement [11].

While useful, the above guidelines were not specifically aimed at coproduction, with limited involvement of young people in developing the recommendations. Those that did involve young people [31], typically drew on the insights from a limited pool of young people working within pre‐established YPAGs. Lastly, none of the guidelines have been developed with longitudinal designs in mind. Arguably, this design has specific demands and guidelines must take this into account. Thus, as noted above, this paper aims to overcome these limitations by (1) involving young people in the development of the MHIM‐YPAG protocol, (2) engaging those from a diverse background and (3) focusing on coproduction for longitudinal designs.

## 5 Evaluation

There is increasing acknowledgement of the importance of evaluating coproduction [7]. Arguably, this should be embedded as early as possible, pertain to specific outcomes, and use measurable indicators. Indicators could include (1) young people's outcomes, (2) research structure/process and (3) wider research outcomes. Recording the impact of a web‐based YPAG, Fernandes et al. [7] reported impacts such as a novel research method, enhanced mental well‐being insights and the meaningful involvement of young people in knowledge‐exchange. Mandoh et al. [20] also reported on the evaluation of their coproduction, noting an increase in young people's leadership skill scores. Finally, Tsang et al. [33] found that young people reported personal growth in general leadership, tangible skillset development and a general improvement in their understanding of the research process.

Despite the above findings, the evaluation of coproduction impact is still in its infancy [7]. Few studies report evaluation information and no standardised methodology for evaluations exists. It has been argued that this produces information blocks that prevent learning [30]. In developing evaluation methods for coproduction, the field could draw on existing PPIE evaluation frameworks such as the GRIPP2 and 4Pi [34, 35]. However, as mentioned by Staniszewska et al. [35], those involved in establishing these frameworks did not include (1) adolescents and (2) those less likely to be engaged in PPI. Thus, this paper will outline an evaluative framework which is coproduced with young people.

## 6 Addressing Challenges of Designing a Coproduction Process

While coproduction best practice guidelines have been invaluable; they leave considerable latitude for defining the specific coproduction process in individual studies. As indicated above, there are a variety of ways of involving young people, however this can be resource intensive and consequently unrealistic for most research budgets. Young people also tend to have busy schedules with numerous competing demands which may lead to feelings of ‘over‐commitment’ [14]. As such, pragmatic choices must often be made to ensure feasibility. Studies with different designs, questions, and goals also require tailored YPAG input that is not reflected in more generalised guidance. For example, longitudinal studies can benefit from sustained involvement of a YPAG over many years and their input on issues that are not relevant for cross‐sectional studies (e.g., retention). Taken together, studies are left with many degrees of freedom when it comes to designing their coproduction process with young people. This protocol, through an engagement with young people, attempts to ensure that these choices reflect the views of those most affected by the research.

## 7 Protocols for Coproduction

Given the need for studies to make a number of decisions about how to involve young people in research, we argue that it is beneficial to create and publish – at a study's outset – a ‘coproduction protocol’. Protocol publication has been associated with numerous benefits in other fields, including systematic reviews, meta‐analyses and trials [36, 37]. As stated, our protocols combine best practice guidelines with input from young people themselves.

A major benefit of protocol publication in a coproduction context is that it promotes commitment to involving young people in ways that are consistent with both best practice guidelines and their stated preferences. It also encourages detailed and systematic a priori planning, which is likely to lead to better quality coproduction. This may be particularly beneficial for supporting the evaluation of coproduction, which requires a clear statement of the aims and careful planning for capturing impact [7]. The opportunity to gain peer review feedback can further strengthen the coproduction process. It is also in line with open science principles and promotes transparency from the very beginning. In coproduction where methods and best practice guidance continue to evolve, the availability of detailed and timely accounts of methods can help enhance our understanding of how to optimise coproduction methods. Therefore, in the current paper, we present a protocol for the coproduction aspect of the MHIM study. This is the anticipated process based on current evidence and young people's input; however, it is important to note that the process will be subject to continual review and feedback by our MHIM‐YPAG. Where changes are made relative to the current protocol because of young person feedback, we will document the changes in future publications.

## 8 Methods

### 8.1 The MHIM Study

The MHIM project is study, funded by the Wellcome Trust, focusing on understanding the interplay of factors influencing adolescent mental health over time. The MHIM project will employ an accelerated cohort design, tracking five age‐based cohorts (11, 12, 13, 14, and 15 years old at the baseline) over 5 years. Participants will complete online surveys and measurement bursts of ecological momentary assessment (EMA) every 6 months. Daily sleep patterns will be monitored using radar‐based technology and hair/finger‐nail samples will be collected at three key measurement points, offering insights into biomarkers (e.g., cortisol) indicative of cumulative stress levels. In addition, parental online surveys will be completed on an annual basis to provide information from multi‐informants.

### 8.2 ‘Pre‐Study’ YPAG

Alongside recent literature, the protocol was informed by a dedicated ‘pre‐study’ YPAG which examined young people's views on coproduction in relation to MHIM and research more generally. We utilised a basic advisory/steering group methodology [38, 39] with advisors recruited via third sector/charitable organisations. To gain as diverse a group of young people as possible, 20 organisations were contacted regarding recruitment (3 agreed to collaborate). The final number of participating young people was 6. These 6 participants were between 11 and 18 years old. 4 identified as female, and 2 as male. Sessions took place online to allow young people from across Scotland (the geographic scope of MHIM) to participate. However, all participating young people were from the central belt of Scotland. It is important to note that this differs from the proposed number of participants in the wider MHIM YPAG (*n* = 18). We did not specify that young people needed to have lived experience regarding mental ill‐health as we view mental health as a topic of relevance for all adolescents; however, as this was advertised as having a mental health focus, we suspected that it would draw those with lived‐experience.

Young people participated in two sessions, the first provided an overview of the project, the research process, advisory panels and coproduction to ensure participants had sufficient knowledge. Young people were then asked 8 questions relating to mental health research and coproduction methodologies. The aim of these questions was to obtain young people's ideas on how to support their continued engagement as coproducers over a 5‐year period. The second was a feedback session. Within this session, the research team provided an overview of the findings from the first and allowed young people to provide comments.

On completion of the pre‐study YPAG sessions, a member of the research team (non‐YPAG member) performed the analysis (thematic analysis (TA); see [40, 41] and created the initial themes. It is important to note that while Braun & Clarke have argued against the dichotomising of TA, the researcher utilised a more semantic‐orientated approach. This was due to the fact that the primary aim was to assess young people's explicit ideas over latent meaning creation. To balance the potential influence of the researcher, the themes, and the associated report, were sent to the young people for further feedback. Young people were asked if they felt these themes matched the discussion and if they would remove/add items. The young people did not ask for any amendments to be made.

The role of the young people in developing the MHIM‐YPAG protocol was akin interactive consultation. The reason for this is that, while young people were engaged in the decision‐making process and their voice was central, they were not involved in its entirety. This differs from the approach we plan for the MHIM‐YPAG, which will be coproduction (see the Supporting Information).

## 9 Results and Protocol Development

The findings that emerged out of the pre‐study YPAG could be thematised into six distinct areas. These pertain to (1) benefits of involving young people in mental health research, (2) outcomes for young people, (3) level of involvement, (4) time commitments/constraints, (5) training/support needs, (6) reflections on applying. A brief overview of these is as follows:

- Involving young people is critical to developing relevant aims, approaches and instruments.
- Regular sessions (every 1–2 months) and communication are needed to support a sense of connectedness to the group and project. This will increase the likelihood of continual engagement.
- Young people think it is important for them to be involved throughout the research process. However, they commented specifically on the importance of idea generation, recruitment and dissemination.
- Participation should reflect the interests, skills and abilities of young people.
- Research needs to be flexible when involving young people due to additional demands within young people's lives, e.g., school.
- Need to facilitate community building, both informally and formally. This includes in‐person sessions and online group building.
- Formal training required to support engagement in different aspects of project.
- Informal support related to the need for researchers to be understanding and accepting.
- While including an application process could pose risks on balance it should be used.
- Involving young people in knowledge‐exchange is important as they use accessible language.

### 9.1 Reflections on the Hierarchy of Involvement: Coproduction

Our pre‐study YPAG findings suggested that young people are interested in taking part in all aspects of the research process. Specifically, they noted interest with three stages: (1) the formative phase, (2) conducting research, e.g., recruitment and (3) dissemination. Young people suggested that they want to be involved as coproducers whereby they are at the core of the decision‐making process. Thus, the below protocol was formulated with this in mind. It is important to note that participation will not be mandatory and thus young people can take part in as many, or as few, sessions as they would like. Given that participants in the pre‐study YPAG noted their scheduling difficulties, flexibility will be essential throughout.

Within the MHIM‐YPAG protocol, coproduction means the close collaboration between young people and researchers in the development and running of the MHIM project. This is not limited to young people supporting the construction of project parameters but also includes their direct involvement in recruitment, data analysis and dissemination [42, 43, 44, 45]. However, due to the flexible nature of involvement, there may be variations in how young people engage. For instance, while they will be involved in analysis and will be provided with appropriate training, the use of advanced statistical methodologies may lead to their involvement being more akin to light consultation. This may also be the case for the creation of academic outputs, e.g., papers. Thus, while coproduction is the overall approach, the specific levels of involvement in aspects of the project will sometimes be constrained by the realities of conducting, at times, highly technical research.

## 10 Protocol Summary: MHIM‐YPAG

Given the pre‐study YPAG findings and recent literature, the following protocol was developed. A detailed protocol is provided in the Supporting Information (see Table S1). Importantly, this is changeable as we will allow MHIM‐YPAG members to shape the protocol as it unfolds. This project received ethical approval from the School of Philosophy, Psychology & Language Studies at the University of Edinburgh.

### 10.1 Recruitment

A total of 18 MHIM‐YPAG members will be recruited based on the expectation that not all members will have capacity at all times. Incentives will include vouchers, educational opportunities, peer network development and leadership qualifications. The MHIM‐YPAG members will be divided into two groups: 11‐13 and 14‐18. Age and UK residency will be the only explicit inclusion criterion. Consent will be obtained from young people and parents/caregivers if under 16. Having a mental health diagnosis will not be a criterion. The reason for this is that (1) mental health is relevant for all adolescents and (2) the wider MHIM study will sample from the general adolescent population. However, we do expect a proportion of those who apply to have some experiences (including a formal diagnosis).

Young people will be recruited through third‐sector organisations and Scottish schools. Alongside more general organisations, those that engage young people from minoritized communities will be contacted, e.g., relating to sexual orientation, ethnicity/race, care experience, neurodivergence/learning disability and socioeconomic status to maximise the chances of achieving a diverse MHIM‐YPAG. Once gatekeeping relationships have been established and a recruitment strategy collaboratively developed, relevant adverts will be distributed.

### 10.2 Application Process

Young people will be asked to apply to be a member of the MHIM‐YPAG. This application will ask them to describe their reasons for involvement. Due to concerns noted by the pre‐study YPAG members, those that apply will be able to do so in a variety of ways, e.g., upload a video, audio file, text etc. Two members of the research team will review the applications to (1) check applicants meet criteria and (2) gauge applicants' motivations.

### 10.3 Sessions

#### 10.3.1 Formative Phase

According to those who took part in the pre‐study YPAG, establishing relationships is key to maintaining young people's continued involvement. Given this, sessions before primary data collection – formative phase – will run every 1–2 months. Within this stage, there will be a mix of **core** and **optional** sessions. The former will consist of four sessions, these include (1) ‘mental health concepts’, (2) ‘mental health measures’ (3) ‘measuring stress (bio‐sampling) and sleep’ and (4) ‘EMA’. These sessions are of critical importance and will form the overall MHIM study structure. In addition, the pre‐study YPAG members emphasised the need for their involvement at this stage.

#### 10.3.2 Data Collection Phase

Once the MHIM data collection period begins, sessions will run every 2 months, again a reflection of pre‐study YPAG comments. Like above, these will be divided into **core** and **optional** sessions. **Core** sessions will include ‘data chat and dissemination strategy’ sessions. The reason that these are core is that pre‐study YPAG members emphasised the importance of their involvement. Sessions classified as **optional** include ‘output’, ‘training’ and ‘informal team building’. Given the length of the study, MHIM‐YPAG members will be informed of the project duration but invited to commit annually. Thus, within each year of data collection, young people will be able to participate in 1–2 core and 2–5 optional sessions – these may fluctuate depending upon training needs, etc. (see the Supporting Information for a further breakdown).

#### 10.3.3 Evaluation

Evaluation for young people will be examined through testimonials and an evaluation survey. An evaluation framework will be developed to assess if young people's aims are met. This framework will be coproduced with young people. Indicators may include relationship development, increased knowledge of mental health/research process, qualification acquisition and research skill development.

Evaluating the effect of involving young people in research is challenging, specifically when developing quantitative metrics [7]. However, there are other ways that evaluation can be achieved, for example, tracking changes associated with involving young people. For instance, when developing research instruments through concept selection, we will examine the benefits of including novel concepts advocated for by young people which would otherwise not have been possible to test. When we develop new instruments, we can assess whether they are of higher quality than others. Regarding recruitment, we will aim to document the extent to which involving YPAG members increased participation. Another method will be to collect qualitative feedback from members about the impact of coproduction on them.

## 11 Discussion

As stated throughout, this paper outlines the MHIM‐YPAG protocol. In addition, informed by relevant literature and pre‐study YPAG insights, it also focuses on developing an evaluative framework for measuring coproduction success. Including young people as coproducers is critical to improving study acceptability, feasibility and appropriateness [46, 47, 48]. Alongside study benefits, there are noted benefits for young people themselves. For instance, Tsang et al. [33] highlighted the positive impacts which YPAG participation had on personal development, specifically improved self‐efficacy, teamwork and leadership. Thus, including young people throughout can be bi‐directionally beneficial. Given this, it is important that young people are involved early [11].

The pre‐study YPAG findings provided invaluable insight into how young people would like to be involved within longitudinal mental health research. While findings relate to MHIM, many have broader relevance. Young people emphasised the importance of involving them within three stages of research: (1) research design, (2) conducting research and (3) knowledge‐exchange. However, they also noted the importance of their involvement throughout. Given this, the protocol allows for engagement flexibility, such that their level of involvement can change. Since a formative principle of this protocol is that it is asset‐based, it is critical that young people feel able to participate when it works for them. This is critically important given the projects length. Yet it is also important to note that this must be balanced with resource constraints. As noted above, a key limitation associated with involving young people within longitudinal studies is associated resource pressures.

Findings from the pre‐study YPAG contributed to the development of a protocol tailored to meet the needs of a longitudinal study. It is important to note that due to the long‐term nature of MHIM the protocol may change to meet coproducer needs/insights. The composition of the MHIM‐YPAG will also likely differ from the pre‐study YPAG consulted in the development of the protocol.

A number of attempts have been made to develop guidelines on engaging young people as coproducers within research [31, 32]. However, they have a number of limitations when applied to specific studies. First, existing guidelines were not developed in relation to longitudinal designs. This protocol rectifies this by engaging young people in a 5‐year coproduction process. Second, these guidelines were rarely developed coproductively and where young people were engaged they typically came from existing, non‐diverse, YPAGs. Again, this protocol attempts to remedy this. While the number of young people involved in the pre‐study YPAG was small, MHIM‐YPAG members will be involved in refining the protocol as it unfolds. This will meet the need for involving a large and diverse group of young people.

There have also been a number of attempts to measure and evaluate the impacts of involving young people. These include those associated with PPIE, such as GRIPP2 and 4Pi. Commenting upon the benefit that this has for research, Fernandes et al. [7] developed a 5‐level evaluative framework by which to do this. In relation to the benefits to young people, Mandoh et al. [20] evaluated impact by assessing changes over a 12‐month period in key indicators: self‐efficacy, leadership skills and collective participation. However, while both strategies provide a method of evaluating impact, it is suboptimal to adopt (1) a generalised evaluative framework not tailored to the needs of a longitudinal project and (2) a generalised framework not coproduced with young people. Thus, there is a need to establish evaluative parameters specific to the MHIM project. This protocol addresses this limitation by tracking study changes made as a result of pre‐study YPAG input. This will be elaborated through the coproduction of an evaluative framework with MHIM‐YPAG members, based on which impacts they feel are most crucial.

While this is currently an underdeveloped area, it may also be interesting to examine the impacts of coproduction on researchers. While there is a tendency to see knowledge‐exchange as unidirectional there is strong potential for this to occur bidirectionally. There has been much work done on this within the wider social science community, specifically within social work and sociology. Within these disciplines there is a focus on researcher reflexivity [49]. This has typically been utilised within qualitative methodologies to (1) promote rigour, reliability and validity [50] and (2) help researchers understand their positionality to undermine power imbalances [51]. Arguably, there is a place for utilising these insights to support the development of bidirectional evaluative indicators.

There are also a number of issues that need to be addressed when recruiting coproducers. Specifically, sample bias due to baseline ‘high skill level’ [20] and the non‐engagement of those who define themselves as outside the ‘inner‐circle’ [14]. These issues arguably lead to the dominance of specific voices/insights, e.g., high achievers and concerns with generalisability. To overcome this, we will recruit from a wide range of sub‐populations.

The publication/pre‐registration of research protocols before undertaking a study has become a common assurance practice within a variety of methodological approaches [36, 37]. The rationale for this is that it provides better transparency and safeguards against un‐robust, post‐hoc, research practices [52]. In addition, it allows for methods to be reproduced to test the robustness/generalisability of findings [53]. Moreover, in relation to the involvement of young people, it allows for the development of standards a priori which could lead to improved quality coproduction and clear expectations from the outset.

A number of authors have commented upon the potential drawbacks associated with the pre‐registration of protocols; however, this has typically pertains to unblinding and ‘contamination’ [54]. However, one pertinent risk in the context of coproduction is that protocol adherence could lead to inflexibility during implementation. As noted above, young people emphasised the need for flexibility and a responsiveness to their views. As such, we anticipate that the protocol will evolve over the course of the project, with changes and their rationale documented in study publications.

## 12 Limitations

A major strength of this protocol is that it was developed with a group of young people. However, as the pre‐study YPAG consisted of six participants, there is a high risk of sample bias and unrepresentativeness. To overcome this limitation with the main MHIM‐YPAG, a number of organisations that represent a wide range of voices shall be engaged. In particular, this will include those that work with and advocate on behalf of minoritized sub‐populations. Currently no standards exist that indicate the optimal number of YPAG participants to ensure minority sub‐populations are represented. We may be able to take insights from qualitative studies, e.g., Hennink and Kaiser [55], who propose 7–17 interviews and 4–8 focus groups. However, if we do, we must take seriously the insights offered by Scholz et al. [21] regarding the meaning of representativeness. It is important to note that this is one part of a broader engagement programme, whereby young people, community organisations, schools and families are engaged.

## 13 Conclusion

There are a number of benefits associated with engaging young people as coproducers within research. However, there is limited knowledge, guidance and standards regarding how this should happen in specifc contexts. In addition, there is very little awareness of how to effectively evaluate the impact/s of involving young people in research. The above protocol – a first of its kind – details the steps that will be taken to coproduce the MHIM longitudinal study, including its evaluative framework.

## Author Contributions

**Luke Power:** writing – original draft. **Tong Xie:** writing – review and editing. **Thomas Bartlett:** writing – review and editing. **Dejla Hoxha:** writing – review and editing. **Heather Bryson:** resources. **Eva Drummond:** resources. **Poppy Fairbairn:** resources. **Alex Swan:** resources. **Yu‐Wen Tan:** resources. **Lorna Caddick:** writing – review and editing. **Aja Murray:** writing – review and editing.

## Conflicts of Interest

The authors declare no conflicts of interest.

## Supporting information

Supporting information.

## Data Availability

The data that support the findings of this study are available on request from the corresponding author. The data are not publicly available due to privacy or ethical restrictions.
