# Supplementary material for: Coproducing an Ecological Momentary Assessment Measurement Burst Mental Health Study With Young People: The MHIM Coproduction Protocol
Source: Health Expect. 2025 Mar 11;28(2):e70218. doi: 10.1111/hex.70218 (PMC11894036; doi:10.1111/hex.70218)
Supplement: Supplementary file 1 — Supporting information. [file HEX-28-e70218-s001.docx]

**Supplementary Materials:**

**S1) Materials from pre-study YPAG**

**Rationale for pre-study YPAG**

A separate pre-study YPAG was developed primarily due to time considerations. While the longitudinal nature of this study provided us with more time than most, we still had constraints. For instance, our start date for the MHIM study required that the YPAG ‘formative phase’ was completed prior to the start dat, allowing for young people’s insights to be effectively integrated into the various study protocols e.g., sleep measurement, stress bio sampling etc. If we had waited until we had recruited all 18 participants for the MHIM-YPAG we would have limited time between developing the MHIM-YPAG protocol, starting the formative sessions, developing the study protocols and then implementing them. Given this time pressure we thought it best to run a pre-study YPAG as early as possible. It is important to note that the flexible nature of the protocol allows for the views of those in the MHIM-YPAG to shape the co-production approach taken.

**Session Questions**

Young people participated in 2 sessions, the first provided an overview of the project, the research process, advisory panels and co-production to ensure participants had sufficient knowledge to participate in the question aspect of the session. Young people were then asked 8 questions relating to mental health research and co-production methodologies. The aim of these questions was to obtain young people’s ideas on how to support their continued engagement as co-producers over a five-year period. The second was a feedback session. Within this session the research team provided an overview of the findings from the first and allowed young people to provide comments

The questions asked in the first session were:

1. What would you want to get out of being involved as a co-producer in a project like this?
2. How much of the research process would you like to be involved in as a co-producer?
3. What part of the research process (outlined above) do you find most exciting?
4. What part of the research process (outlined above) do you find least exciting?
5. Do you think there are barriers to being involved as a co-producer in a 5-year project? If so, what are they and how could they be overcome.
6. What would be a reasonable time commitment per year? Given that this is a 5-year project.
7. What kind of training or support do you think you would want to receive to facilitate your involvement/ help you get the most out of the experience?
8. How do you feel about having to apply for to be a part of a YPAG?

It is important to note that these questions acted as a guide and consequently young people engaged in this aspect of the session commented upon a variety of issues outside of these questions. All of which are reflected in below protocol.

**S2) Detailed Protocol**

**Co-production goals**

The goals of the co-production process can be divided into two types of goals: 1) benefits for the research and 2) benefits for the young people involved.

**Benefits for research**

- Increased acceptability, appropriateness and feasibility of MHIM study.
- Support with recruitment.
- Greater relevance of the research project to issues that are important to young people.
- Potential for bidirectional knowledge exchange, with researchers supported to develop new practices associated with engaging young people.
- Improved dissemination.

**Benefits for Young People**

- Provide young people with a sense of supportive community.
- Provide young people with a sense of achievement in the knowledge that they are having an impact on mental health research and practice.
- Provide young people with knowledge, skills and career/personal development opportunities in areas based on their stated preferences. This includes offering qualifications (e.g., Participative Democracy Certificate- SCQF level 5), providing training sessions on specific topics or skills (e.g., mental health knowledge, presentation skills, research skills), providing networking opportunities (e.g., meeting with peers, mental health researchers), providing opportunities to gain or develop skills and experiences (e.g., opportunities to contribute to data collection or analysis, publications, presentations and reports). Financial compensation: £30 voucher for each online session. Compensation amount was based on NIHR guidelines (NIHR 2024). If they take part in an in-person session their travel/food will be compensated and they will receive a £30 voucher. This also factors in the limited work they may need to undertake in preparation for the session. In addition, if they are attending a training session the training opportunity will be classified as the benefit.

**Co-Production Process**

*Principles*

The guiding principles derive from insights made by young people involved in the pre-study YPAG. It is interesting to note that there is much overlap with those developed by organisations such as INVOLVE/ The New Economics Foundation and those commented upon by the pre-study YPAG. These principles include (1) building/ maintaining relationships, (2) the importance of including a variety of perspectives, (3) an asset-based approach which builds on existing capabilities (4) respecting/valuing the knowledge of all, (5) power sharing (6) flexibility and understanding. Given these similarities, this protocol will adopt these six guiding principles.

*Training for Young People*

Young people expressed their interest in being directly involved in the research process e.g., collecting and analysing data. This consequently necessitates making training available to them so they may effectively do this. Accordingly, a number of training sessions will be set up to enable this. These sessions will cover topics such as research strategy, bio-sampling (collecting and analysing), creating and interpreting data outputs, report writing and presentation skills. The research team will be able to meet these training needs.

*YPAG Recruitment*

Previous YPAG members have reported that opportunities to take part in YPAGs may feel inaccessible to those who do not have links to the relevant research communities, e.g., a relative who works at a university (Watson et al., 2023). To ensure wider representation within in the MHIM-YPAG, we are working with organisations who support typically under-represented groups to advertise the opportunity widely.

*Recruitment Process and Sessions*

A total of 18 MHIM-YPAG members will be recruited. The reason that this is to (1) keep the number small enough to enable the development and consolidation of relationships and (2) big enough so that we had a diverse range of views included. The MHIM-YPAG members will be divided into two groups: the younger group will have an age range of 11-13 at baseline and the older group will have an age range of 14-18 at baseline. Those organisations which have the capacity to support recruitment will be asked to advertise this opportunity though their various networks of professionals and young people. Young people will be asked to email the study address if interested. If they do, they will be asked to apply to be a member on the MHIM-YPAG. This application will ask them to describe the reasons that they wish to be involved. In addition, due to concerns noted by the pre-study YPAG members regarding the application process, those that apply will be able to do so in a variety of ways e.g., they will be able to upload a video, audio file, text etc. Applicants will be given a date which they will have to apply by. Two members of the research team will review the applications. The applicants will be assessed in order to (1) assure applications meet the criteria (between ages 11-18) and (2) gauge applicants understanding regarding mental health awareness.

Young people will be recruited for the MHIM-YPAG through an engagement with a wider range of organisations. The reason for this is that by engaging with a variety of community organisations that work with young people we are able to hear from a diverse range of young people. This is particularly important when we consider the need to involve those from underrepresented communities. Given this, our recruitment strategy will specifically focus on those organisations that work with young people from underrepresented communities e.g., those classified as ethnically minoritized, neurodivergent, care experienced, impoverished.

Session will be broken up into two primary clusters: **‘Formative’** and **‘Data Collection’**. Within these there will be a mix of **core** and **optional** sessions. It is important to note that not all young people will be at every session. Young people stressed the need for flexible participation due to additional demands and constraints on their time. In addition, they commented upon creating space for peer-to-peer rapport building, thus emphasising the need for non-research sessions (**optional**). It is expected that within each session there will be at least 5 young people from each age group (n=10). In addition, given that young people may be reluctant to commit to a full five years of the project, MHIM-YPAG members will be informed of the 5-year duration but invited to commit to one year of the project at a time. Thus, within each year, young people will be able to participate in 2 core sessions and 2-5 optional sessions (these may fluctuate depending upon training needs and dissemination sessions).

Within these sessions, young people will be involved in collaboratively making decisions regarding the overall structure of the project and its direction. In relation to the protocol’s formative stage, young people will be involved in shaping the approach taken, this will specifically pertain to deciding (1) which mental health concepts and measures should be included (2) the protocol for measuring for both stress and sleep and (3) the overall structure of the EMA and survey. In relation to this later point, as there are limited EMA measures validated, specifically those pertaining to relationships, we will co-produce these with our MHIM-YPAG members. To show how young people will be involved in the decision-making process, the example of mental health concepts can be used. Members of the MHIM-YPAG will be asked to discuss different mental health concepts and their appropriateness for the project. Prior to the start of this session young people will be given an accessible document detailing various mental health concepts. They will be asked to think about the relevance of these concepts, and others like them, for their own mental health. Within the session they will discuss these mental health concepts, and any others which they consider pertinent but were not included in the breakdown. After this discussion they will then be asked to vote on which concepts should be included. It is important to caveat this by stating that there are some constraints as to which measures will be included e.g., due to mandates from the funder. This will be made clear to the YPAG.

It is important note that for each session there will be a decision which needs to be made, as shown within the examples above. This will be done by each participant, including those on the research team. All votes will hold the same weight and the final outcome will be based on majority. The reason for this is that this aims to undermine the inherent power differential within this dynamic.

Alongside making decisions young people will be directly involved in undertaking the project. For instance, young people will have key recruitment responsibilities such as developing posters, creating social media content, contacting schools, giving presentations to teachers/pupils etc. Young people will also have responsibilities related to other aspects of the study, responsibilities will include e.g., supporting the examination of data analysis, writing reports collaboratively and presenting findings to key stakeholders. It is important to note that this is something which, through our pre-study YPAG, young people expressed their interest in doing. Finally, MHIM-YPAG members will be able to influence/ change the protocol as it unfolds.

*Frequency, Format and Cadence of Sessions*

Bi-monthly meetings will take place during the formative phase of the project to allows for community development. Once the data collection phase starts this will go down to every six months. These sessions will primarily focus on findings and their dissemination. However, optional sessions will be run in between these more focused sessions which focus on team building and/or skill development.

Considering the complementary benefits and drawbacks of in-person versus online meetings, meetings will be a mixture of online and in-person. In-person meetings will take place at the University of Edinburgh and last around 5 hours, including a 1/1.5-hour break. Young people who took part in the pre-study YPAG and are currently preparing for higher education, expressed an interest in doing this as they want to experience university life. In addition, young people stated their desire to meet and network with peers. The number of in-person session will depend on available resources, however we plan to have 6 throughout the entire project Online sessions will use MS Teams, the platform preferred by the majority of young people. In addition, online sessions will run between 90-120 mins to allow time for research discussion and rapport-building. Some work may be required prior to the sessions to allow young people to familiarise themselves with session content. For example, pertaining to mental health concepts, young people will be given a breakdown of these concepts, including examples, to support familiarisation prior to the session.

**MHIM-YPAG Community Building**

Young people that took part in the pre-study YPAG noted that one of the reasons they wished to participate was to meet new people. They highlighted the influence this would have on their desire to continue engagement. It may be argued that, a key method of preventing attrition is through the nurturing of these relationships and the subsequent development of a study culture. This study culture creates an environment were all feel connected to and apart of the project. According, this shall be done by (1) building collective principles/ground rules, (2) running informal social events and (3) supporting the development of non-research communication.

- 1. MHIM-YPAG participants will co-create a set of collective principles and ground rules through facilitated discussions in the first few sessions. The principles/rules will cover aspects such as mutual respect, confidentiality, acknowledgement of diverse perspectives. Through this consensus-forming process, we hope to ensure a safe, respectful and inclusive environment. In addition, it is hoped that this will encourage MHIM-YPAG members to foster a sense of ownership and responsibility over the project.
  2. Organise occasional informal social events or activities to strengthen a sense of community among researchers and MHIM-YPAG members. These events can provide opportunities for team bonding and relationship-building outside the formal research setting.
  3. Encourage informal communication channels for researchers and young people to permit connectivity outside of formal research discussions. This could include allocating a brief period in every session, creating a dedicated chat group on social media, or other channels for sharing updates and experiences and keeping in touch between meetings.

**YPAG Sessions: type and content**

Young people will be involved in key decisions throughout the MHIM project. These decisions will include finalising (1) the inclusion of mental health concept and their associated measures; (2) stress and sleep protocol; and (4) the dissemination strategy. Table S1 provides a breakdown of each session over the 5-year period. Other aspects/activities associate with the research process that MHIM-YPAG members will may be involved in, beyond their input at meetings, include:

- Making videos for the website
- Reviewing and contributing to study materials
- Recruitment
- Data analysis
- Delivering presentations
- Co-authoring articles
- Being involved in other outputs like podcasts/ writing blogs etc.

**Table S1: Content of YPAG Sessions over 5-year MHIM Study**

| **Session** | **Content** |
| --- | --- |
| **Session 1 (N/A):** Introduction and Session Sign-up | Ice breakers; introduction of participation and staff; discuss PDC and method of signing up; developing guidelines and standards; ascertain what young people wish to get out of participating in sessions and as co-producers, supporting the development of evaluation framework. |
| **Session 2 (Core):** Mental Health Concepts | Overview of mental health concepts; young people to rank them; young people to decide which concepts are appropriate for use in MHIM study, including any not within the predefined list; finalise concept list. |
| **Session 3 (Core):** Mental Health Measures | Overview of mental health measures (associated with session 2); young people thoughts on measures presented; young people to decide which measures are appropriate to be used in MHIM project; vote on final preferred measures for each concept. |
| **Session 4 (Optional- In-person and online):** Measuring Stress and Sleep sampling | Overview of measuring stress (bio-sampling e.g., hair/nail) and sleep including why and how it is done; young people will be shown how this is done and given an opportunity to do it themselves; aim is to ascertain whether or not young people think this is viable and, if so, how it should be undertaken; finalise protocols for measuring both stress and sleep. |
| **Session 5 (Core- online):** EMA and Measure Development | Overview of ecological momentary assessment tool. Young people will be asked their opinions regarding its use e.g., potential barriers; young people to partake in an exercise to develop a relationship EMA measure |
| **Session 6 (Core-online):** Feedback on Pilot and Overall Design | Young people provide feedback on survey/EMA after piloting. Provide opportunity for young people to develop list of bugs/ concerns needing attention. |
| **FIRST YEAR OF DATA COLLECTION** | |
| **Session 7 (Optional):** Training Needs | Team will reiterate the different aspects that young people can be involved in; young people work with us to establish what training would be needed. |
| **Session 8 (Core- In-person):** Data Chat and Dissemination Strategy | Overview of findings from first wave of data; young people to provide comments and help prioritise research questions; YP discuss possible dissemination. Overview of the ways data is disseminated; young people thoughts on dissemination e.g., platforms used/ organisations/ individuals contacted; means of dissemination (written, video, audio); development of a step-by-step breakdown of recruitment. |
| **Session 9 (Optional):** Informal Team Building | TBC (by young people) |
| **Session 10 (Optional):** Training | Research strategies |
| **Session 11 (Core):** Data Chat and Dissemination Strategy | Overview of findings from second wave of data; young people to provide comments. YP discuss possible dissemination. |
| **Session 12 (Optional):** Output | Young people to present findings to group/s of young people participating in the project |
| **SECOND YEAR OF DATA COLLECTION** | |
| **Session 13 (Optional):** Training | Bio-sampling/ hair/nail-sampling |
| **Session 14 (Core- in-person):** Data Chat and Dissemination | Overview of findings from third wave of data; young people to provide comments; young people discuss potential dissemination |
| **Session 15 (Optional):** Informal Team Building | TBC (by young people) |
| **Session 16 (Optional):** Training | Data interpretation and visualisation |
| **Session 17 (Optional):** Training | Presentation skills |
| **Session 18 (Core):** Data Chat and Dissemination | Overview of findings from fourth wave of data; young people to provide comments. Young people to discuss potential dissemination. |
| **Session 19 (Optional):** Output | Young people to present findings to group/s of young people participating in the project |
| **THIRD YEAR OF DATA COLLECTION** | |
| **Session 20 (Optional):** Training | Writing skills |
| **Session 21 (Core- In-person):** Data Chat and Dissemination | Overview of findings from fifth wave of data; young people to provide comments. Young people to consider and develop strategy for third year dissemination. |
| **Session 22 (Optional):** Informal Team Building | TBC (by young people) |
| **Session 23 (Optional):** Training | Data analysis |
| **Session 24 (Core):** Data Chat and Dissemination. | Overview of findings from sixth wave of data; young people to provide comments. Young people to discuss potential dissemination. |
| **Session 25 (Optional):** Output | Young people to present findings to group/s of young people participating in the project |
| **FOURTH YEAR OF DATA COLLECTION** | |
| **Session 26 (Optional):** Informal Team Building | TBC (by young people) |
| **Session 27 (Core- In-person):** Data Chat and Dissemination | Overview of findings from seventh wave of data; young people to provide comments. Young people to consider and develop strategy for fourth year dissemination. |
| **Session 28 (Optional):** Training | (young people to decide) |
| **Session 29 (Core):** Data Chat and Dissemination | Overview of findings from eighth wave of data; young people to provide comments. Young people to discuss potential dissemination. |
| **Session 30 (Optional):** Output | Young people to present findings to group/s of young people participating in the project |
| **FIFTH YEAR OF DATA COLLECTION** | |
| **Session 31 (Optional):** Informal Team Building | TBC (by young people) |
| **Session 32 (Core- In-person):** Data Chat and Dissemination | Overview of findings from ninth wave of data; young people to provide comments. Young people to consider and develop strategy for fifth year dissemination |
| **Session 33 (Optional):** Informal Team Building | TBC (by young people) |
| **Session 34 (Core):** Data Chat and Dissemination. | Overview of findings from tenth wave of data; young people to provide comments. Young people to discuss potential dissemination. |
| **Session 35 (Optional):** Output | Young people to present findings to group/s of young people participating in the project |

Table S1: Content of each YPAG session over 5-year period. Those items with content noted TBC will be decided with young people.

**Reporting**

*Reporting in Project Outputs*

To make it clear how the research has been shaped by young people’s input, research publications will include a **summary of the young people contributions**, including specific components that were included or changed as a result of young people input. In addition, **MHIM-YPAG members will be co-authors on all papers** (with their consent) where co-production has played a significant role. Since some young people may prefer to remain anonymous, young people will be given a choice whether to be acknowledged by name or as part of the MHIM team. Lastly, research reports will include information about the MHIM-YPAG, **following YPAG reporting guidelines** developed by earlier authors (e.g., Sellars et al., 2020). This will include information about age range, number of young people, relevant characteristics (e.g., lived experience of mental health issues), as well as the process of engagement. Lastly, young people will be directly involved in producing outputs e.g., articles, reports etc.

*Reporting to the MHIM-YPAG*

Given the importance of demonstrating to young people how their input impacts the research, we will include regular reports to the YPAG on their input. At each YPAG meeting there will be a standing agenda item on the outcomes of the previous sessions i.e., how the input from the last meeting fed into project changes/developments.

**Evaluation**

Evaluation for the involving of young people (MHIM-YPAG) will be examined through testimonials and through an evaluation survey. An evaluation framework will be developed in order to assess whether or not the aims set out by MHIM-YPAG members are met. This framework will be developed through an engagement with MHIM-YPAG members. The development of this evaluation framework has already begun, given the findings noted above. Indicators which may be included relate to relationship development, increased knowledge of mental health and the research process, qualification acquisition.

In relation to evaluating the impact of co-production for research, there is noted difficulty in testing/ evaluating the impacts which young people have on research projects. One of the ways in which this can be done is by tracking changes associated with the involvement of young people. For instance, in relation to developing research instruments through concept refinement, one may be able to examine the benefits associated with including concepts advocated for by young people compared to those suggested by researchers. Lastly, by intricately tracking these changes and subsequently making them available to others, the protocol can be replicated and findings compared. While there are issues associated with these suggestions, they will provide some insight into the impacts associated with young person involvement.
